# Uncovering the architecture of selection in two *Bos taurus* cattle breeds

**DOI:** 10.1111/eva.13666

**Published:** 2024-02-22

**Authors:** Troy N. Rowan, Robert D. Schnabel, Jared E. Decker

**Affiliations:** ^1^ Division of Animal Sciences University of Missouri Columbia Missouri USA; ^2^ Genetics Area Program University of Missouri Columbia Missouri USA; ^3^ Department of Animal Science University of Tennessee Institute of Agriculture Knoxville Tennessee USA; ^4^ Department of Large Animal Clinical Sciences, College of Veterinary Medicine University of Tennessee Knoxville Tennessee USA; ^5^ Institute for Data Science and Informatics University of Missouri Columbia Missouri USA

**Keywords:** cattle, polygenic selection, selection mapping, sweeps

## Abstract

Directional selection alters the genome via hard sweeps, soft sweeps, and polygenic selection. However, mapping polygenic selection is difficult because it does not leave clear signatures on the genome like a selective sweep. In populations with temporally stratified genotypes, the Generation Proxy Selection Mapping (GPSM) method identifies variants associated with generation number (or appropriate proxy) and thus variants undergoing directional allele frequency changes. Here, we use GPSM on two large datasets of beef cattle to detect associations between an animal's generation and 11 million imputed SNPs. Using these datasets with high power and dense mapping resolution, GPSM detected a total of 294 unique loci actively under selection in two cattle breeds. We observed that GPSM has a high power to detect selection in the very recent past (<10 years), even when allele frequency changes are small. Variants identified by GPSM reside in genomic regions associated with known breed‐specific selection objectives, such as fertility and maternal ability in Red Angus, and carcass merit and coat color in Simmental. Over 60% of the selected loci reside in or near (<50 kb) annotated genes. Using haplotype‐based and composite selective sweep statistics, we identify hundreds of putative selective sweeps that likely occurred earlier in the evolution of these breeds; however, these sweeps have little overlap with recent polygenic selection. This makes GPSM a complementary approach to sweep detection methods when temporal genotype data are available. The selected loci that we identify across methods demonstrate the complex architecture of selection in domesticated cattle.

## 1 INTRODUCTION

Since their initial domestication in the Fertile Crescent ~10,500 years ago (Bradley et al., 1996) cattle have been exposed to intense selection for increased tameness, production, and fecundity leading to substantial changes in these phenotypes. While selection occurs on phenotypes, and very recently on estimated breeding values, the phenotypic changes of a population are due to changes in the genotype frequencies of the trait's underlying causal variants. Occasionally, the selection of beneficial mutations of a very large phenotypic effect can lead to very rapid changes in allele frequency at a locus. The resulting “selective sweep” not only increases the frequency of the beneficial variant but also reduces genetic variation in the genomic region surrounding the selected locus (Smith & Haigh, 1974). In cattle populations, multiple sweeps have been mapped to genomic regions, many of which are related to simple Mendelian traits such as polledness (the absence of horns) (Ramey et al., 2013) and coat color (Boitard et al., 2016) or large‐effect quantitative trait loci (QTL) influencing production traits (Gutiérrez‐Gil et al., 2015).

While sweeps at Mendelian loci have played an important role in the domestication and subsequent improvement of cattle populations, it is becoming increasingly apparent that the majority of both historical and ongoing selection is on highly complex traits (Kemper et al., 2014; Zhang et al., 2020). Complex traits are controlled by many mutations of relatively small effect spread throughout the genome (Mackay et al., 2009). Under complex trait architectures, selection can generate substantial changes to a phenotype without necessarily generating large allele frequency shifts (Barghi et al., 2020). These modest directional allele frequency changes at selected loci make mapping selection on polygenic traits over short timescales difficult. However, by leveraging large commercially generated datasets that include influential founder individuals, cattle populations offer intriguing opportunities for mapping the loci exposed to polygenic selection over time (Decker, 2015). The Generation Proxy Selection Mapping (GPSM) method uses a proxy for the number of meioses that separate an individual from the beginning of the pedigree as the dependent variable in a genome‐wide linear mixed model to detect significant associations between generation and allele frequency caused by selection (Decker et al., 2012; Walsh & Lynch, 2018). When pedigrees have missing data or complex, overlapping generations a proxy for generation number, such as birth date, can improve the analysis. Both simulations and empirical data show that GPSM is effective at detecting subtle ongoing shifts in allele frequency across the genome of multiple populations (Rowan et al., 2021).

Genome‐wide association studies have motivated extensive work attempting to dissect the genetic architecture of complex traits in many species (Timpson et al., 2018). We expect that the selection of these complex traits occurs on similar polygenic architectures (Barghi et al., 2020). Here, we expand on previous work exploring the selection landscape in cattle with one of the largest non‐human selection mapping datasets explored to date. By using sequence‐imputed genotypes for over 124,000 individuals in two beef cattle populations, we substantially increase our power to identify subtle ongoing shifts in allele frequency due to selection at over 11 million sequence variants. With these high‐resolution data, we can discern the underlying genomic loci on which selection acts in cattle populations. Further, knowledge of ongoing selection in the bovine genome can serve as important annotations that add context to genomic regions identified in other studies. Additionally, we use subsets of these data to explore recent and ongoing selective sweeps using both haplotype and site frequency spectrum (SFS)‐based approaches.

The genetic architecture of a trait within a population describes the number, mode of inheritance, effect sizes, and distribution of the genetic variants influencing the trait. Here we coin the term “selection architecture” to describe the combination of hard sweeps, soft sweeps, and polygenic selection that have altered the sequence diversity within a population. This combination of selection mapping approaches provides a detailed report of the genetic architectures on which directional selection acts in cattle populations and a blueprint for leveraging temporally distributed genotypes to understand the selection architectures of other species.

We analyze data from two breeds, Red Angus and Simmental from the United States. The Red Angus Association of America was formed in 1954 by breeders creating a new breed association focused on performance records using Angus cattle that were red rather than black (https://redangus.org/about‐red‐angus/history/). The Red Angus Association has a history of best‐practice adoption and has focused on maternal efficiency and fertility. The American Simmental Association was founded in 1968 (https://simmental.org/site/userimages/History%20of%20the%20Simmental%20Breed.pdf). Breeders created American Simmental cattle by importing piebald bulls of various European breeds (Simmental, Montbeliard, Fleckvieh, etc.). These bulls were bred to cows typically of British breed ancestry, and “purebred” Simmental (87.5% Simmental by pedigree ancestry) were bred up by repeatedly backcrossing to Simmental bulls. A relatively small number of full‐blood Simmental are registered in the American Simmental Association herdbook. The American Simmental Association herdbook remains open and animals outside of the Simmental breed can still be used to create animals that are registered in the herdbook. Simmental and Angus animals are frequently mated to create hybrid animals, referred to as SimAngus. Due to the success of the Certified Angus Beef branded program, Simmental cattle have been selected to be polled, black, and solid colored (not piebald). In the United States, each independent beef farm or ranch has their own goals, and these are rarely described in formal, explicit breeding objectives. We investigate the selection architecture (collection of hard sweeps, soft sweeps, and polygenic selection) to reconstruct the history of selection objectives in these two breeds.

## 2 METHODS

### 2.1 Genotype data and imputation

We used commercially generated assay genotypes from two populations of *Bos taurus* beef cattle. An Animal Care and Use Committee protocol is not necessary for this project as DNA samples and other metadata were collected as part of routine animal production practices. These data, made up of assays ranging in density from 25 to 777 K SNPs were filtered, phased, and then imputed using the approach described in Rowan et al. (2019). Briefly, prior to phasing and imputation, we removed individuals with low call rates (<0.90) and SNPs with low call rates (<0.90) or extreme Hardy–Weinberg *p*‐values (<10^−50^, indicative of genotyping errors) in PLINK (version 1.9) (Purcell et al., 2007). Genotypes were phased using a high‐density reference in Eagle v2.4.1 (Loh et al., 2016) and imputed using Minimac4 (Das et al., 2016). The resulting high‐density chip‐imputed dataset contained 811,967 autosomal SNPs for 90,580 registered Simmental and 46,454 registered Red Angus animals. We refer to this dataset as 811 K throughout. High‐density imputed genotypes were then imputed to 43,214,290 SNPs from whole‐genome resequencing data using 9871 reference individuals from the Thousand Bulls Project (Hayes & Daetwyler, 2019). Of these sequenced individuals, 14 Red Angus and 32 Simmental animals were also in our genotyped dataset. We restricted the imputation reference to high quality (Variant Quality Score Recalibration (McKenna et al., 2010) Tranche 90), biallelic variants with minor allele counts greater than 20. Following imputation, we further filtered imputed SNPs, keeping variants with software‐calculated imputation *R* ^2^ (>0.4) and minor allele frequency (>0.01), leaving 14,396,771 and 13,874,618 imputed sequence variants for Red Angus and Simmental datasets, respectively. Genomic coordinates for all array and sequence genotypes were based on positions in the ARS‐UCD1.2 assembly (Rosen et al., 2020). We used both software‐calculated (for all variants) and empirical (for 811 K genotyped variants) *R* ^2^ values to evaluate dataset‐wide imputation accuracies.

### 2.2 Generation proxy phenotypes

We used the breeder‐reported birth date to calculate the continuous number of years (months and days expressed as a decimal) between the animal's birth date and October 19, 2020. This value was used as the continuous generation proxy in GPSM. In addition, due to the extreme left‐skewness of animal birth dates in the dataset, we performed a Box‐Cox transformation of animal birth date to test the effects of transforming the data for normality. For the Red Angus population, we chose 2012 as an arbitrary cutoff date for our “young” dataset. This allowed us to maintain the majority of samples while removing the majority of samples with inflated residuals.

### 2.3 Generation proxy selection mapping (GPSM)

Generation proxy selection mapping uses an individual's generation number or a proxy for generation number as the dependent variable in a genome‐wide linear mixed model. To control for shared ancestry between individuals and to estimate variance components we used autosomal SNP markers in our 811 K imputed dataset with MAF > 0.01 to construct a genomic relationship matrix (GRM) with the method in Yang et al. (2011) for each population. Variance components were estimated using a genomic restricted maximum likelihood (GREML) approach implemented in GCTA (version 1.92.3) (Yang et al., 2010, 2011). To evaluate the impact of transformations to generation proxy phenotypes, we predicted random genetic effects (breeding values) and residuals for individuals using GCTA's “‐‐reml‐pred‐rand” function.

The model used for selection mapping was as follows:

y=μ+bx+a+ϵ

Here, y is a vector of animal generation numbers or generation proxies, μ is the sample mean, bx is the scalar regression coefficient b on an *N* length vector of animal genotypes x, a is a random vector of polygenic terms $\sim N\left(0,G\sigma_{g}^{2}\right)$ where G is a genomic relationship matrix, and e is a random error term $\sim N\left(0,I\sigma_{e}^{2}\right)$. All GPSM analyses were performed using the “‐‐mlma” function in GCTA. When testing the impact of generation proxy transformations on statistical power, we used 811 K imputed genotypes. We performed GPSM on sequence‐imputed genotypes on four total datasets with GRMs calculated with 811 K genotypes.

To further refine GPSM signals and detect additional associations, we performed a within‐analysis conditional and joint analysis (COJO) (Yang et al., 2012) in GCTA (v 1.92.3). COJO utilized summary data and genotypes from each of our sequence‐level GPSM runs. The COJO model was conditioned on SNPs with GPSM *p*‐values < 10^−5^. We controlled for SNP collinearity by setting conditional *p*‐values of highly correlated variants (*r* ^2^ > 0.9) to 1. Significant COJO SNPs were those with conditional and genome‐wide *p*‐values < 5 × 10^−8^.

### 2.4 Haplotype‐based scans for selection

To map genomic regions that underwent strong selection in the distant‐to‐intermediate past, we used the number of segregating loci (nSL) method (Ferrer‐Admetlla et al., 2014) on phased haplotypes from our full Red Angus and Simmental 811 K datasets (MAF > 0.01), as well as on a subset of purebred Simmental animals. Since nSL is an entirely window‐based statistic, we did not want to introduce any additional imputation errors and thus analyzed only 811 K density datasets. The nSL statistic is related to iHS but is more robust and powerful (Ferrer‐Admetlla et al., 2014). The nSL statistic was implemented in selscan (Szpiech & Hernandez, 2014) where per‐SNP scores were also normalized in 100 frequency bins. As opposed to calculating significance in fixed windows, we fit a smoothing spline for each chromosome over normalized nSL scores using the GenWin R package (Beissinger et al., 2015). This allowed us to define variable‐length windows in which we calculated the mean nSL scores. We considered the top 0.5% of these windows that contained at least three SNPs to be significant outliers for downstream annotation and analysis.

### 2.5 Composite scan for selection

We performed composite scans for selection using called sequence genotypes from 14 American Red Angus and 32 registered Simmental animals, some of which may be crossbred, in the 1000 Bull Genomes Project (Hayes & Daetwyler, 2019) in RAiSD (v2.9) (Alachiotis & Pavlidis, 2018). RAiSD calculates a composite selection statistic, μ, aimed at detecting various signatures of selective sweeps. To preserve the full site frequency spectrum, we did not filter on any quality or frequency‐based metrics and used only resequenced animals as opposed to imputed genotypes. Rather, we restricted our analysis to only biallelic SNPs. SNPs in the top 0.05% of RAiSD μ‐values were considered significant outliers as in Alachiotis & Pavlidis, 2018.

### 2.6 Tajima's *D* scores

We used VCF‐kit (Cook & Andersen, 2017) to calculate Tajima's *D* (Tajima, 1989) scores across the genome for Red Angus animals in the 1000 Bull Genomes project. We calculated these scores in fixed‐width 100 kb bins across the genome.

### 2.7 Gene & QTL annotation and enrichment

We annotated nearby genes and QTL using the GALLO R package with gene lists from ENSEMBL version 103 annotations for the ARS‐UCD 1.2 (Yates et al., 2020) and known *Bos taurus* QTL curated in the Animal QTL Database as of October 2020 (Hu et al., 2019). We annotated all genes and QTL within 50 kb of significant (*p* < 5 × 10^−8^) COJO SNPs or sweep outlier regions (based on lead SNP for RAiSD or physical center of nSL window). We performed gene set enrichment analyses on GPSM candidate genes and genes within selective sweep regions using the gost function in the R package gprofiler2, version 0.2.2 (Kolberg et al., 2020). Significant sets were identified as those with FDR‐corrected *p*‐values below 0.1.

## 3 RESULTS

### 3.1 Quantitative genetic signals of polygenic selection

In each population, we used genomic restricted maximum likelihood (GREML) (Yang et al., 2010) to estimate the proportion of variance explained (PVE) by 811,967 imputed SNPs in various subsets of our Simmental (SIM) and Red Angus (RAN) datasets (Tables S1 and S2). Using an individual's date of birth as a generation proxy, we estimated the PVE to be 0.523 (SE = 0.007) and 0.619 (SE = 0.005) in RAN (*n* = 46,454) and SIM (*n* = 78,787), respectively. Results from simulations in our previous work suggest that PVE is mostly a function of the demographics of a population and the strength of selection occurring (Rowan et al., 2021). Due to the non‐normality of sampled birth dates in both datasets (Figure S1), we observe a large divergence from expectation in individual breeding values and residuals in GREML analyses, particularly for the earliest‐born individuals (Figure S2). Although linear mixed models are robust to model misspecification (Visscher et al., 1996; Zhou et al., 2013), we explored transformations to increase our power. To help normalize residuals and potentially boost our power to detect selected variants, we performed a Box‐Cox transformation to birth date (Tables S1 and S2). When using Box‐Cox‐transformed birth date as the dependent variable in Red Angus, PVE increased to 0.657 (SE = 0.006). A Box‐Cox transformation to birth date in Simmental decreased PVE to 0.605 (SE = 0.005). This transformation noticeably normalized GREML‐estimated residuals and breeding values in both datasets (Figure S3).

We explored the effects of various statistical transformations on our ability to detect GPSM signatures in Red Angus using the 811 K SNP dataset. In Red Angus, we observed an increase in the number of variants detected at multiple significance thresholds (nominal: *p* < 10^−5^, Bonferroni: *p* < 7.55 × 10^−7^, and FDR‐corrected *q*‐values <0.1 and <0.05). At all significance thresholds, Box‐Cox transformed birth date detected between 71% and 89% more significant SNPs and at least 12 additional significant loci (Table S3). Interestingly when analyzing data only for animals born since 2012, GPSM identified almost all of the same loci as did the Box‐Cox transformed birth date GPSM on the full Red Angus dataset. This result agrees with simulations from Rowan et al. (2021) that showed that more uniform genotype sampling across generations increased the power of GPSM to detect selection when sample size was held constant. The same transformation applied to the Simmental dataset led to a reduction in the number of identified SNPs and loci, compared with using raw birth date (Table S4).

While the Red Angus dataset was composed of almost entirely purebred individuals, the Simmental dataset contained large numbers of crossbred animals. The number of animals with non‐Simmental ancestry that have been genotyped by the breed association has significantly increased in recent generations (Figure S4). Consequently, we divided the Simmental dataset into subsets based on pedigree‐reported ancestry and/or birth date. This allowed us to examine the selection that is occurring within different subsets of the population. A principal component analysis identified that pedigree ancestry appeared to be supported by genetic relationships in the data (Figure S5). The PVE for birth date remained moderately high, ranging from 0.619 (SE = 0.005) for all animals with >5% SIM ancestry to 0.436 (SE = 0.021) for animals born before 2008. A complete accounting of variance components estimated from these subsets is in Table S2.

### 3.2 Imputation accuracy evaluation

We used multiple metrics to evaluate the accuracy of sequence‐level imputation. The average empirical *R* ^2^ values (accuracy of imputation at 811 K known sites) were >0.99 for both Simmental and Red Angus animals. As expected, imputation accuracy declined at low minor allele frequencies, but even rare SNPs (MAF < 0.01) were imputed with high accuracy (Simmental empirical *R* ^2^ = 0.91 for 96,409 SNPs and Red Angus empirical *R* ^2^ = 0.89 for 129,894 SNPs). We also compared empirical *R* ^2^ values with Minimac's internally estimated imputation quality metrics. These values were moderately correlated for typed variants that had empirical accuracy measurements (*r* = 0.684). When removing sites that were identified as likely poorly imputed (*R* ^2^ < 0.4), the average predicted accuracy was 0.835 for all variants and 0.938 for variants with MAF > 0.01 (14,396,771 for Red Angus and 13,874,618 for Simmental). Taken together, this represents generally high‐quality imputation across both populations.

### 3.3 Generation proxy selection mapping in Red Angus

Based on results from the 811 K GPSM analyses in Red Angus using birth date as the dependent variable, we performed three separate sequence‐level analyses (11,759,568 imputed SNPs) for all individuals, for animals born before 2012, and animals born after 2012, referred to hereafter as full Red Angus, old Red Angus, and young Red Angus, respectively.

**FIGURE 1:**
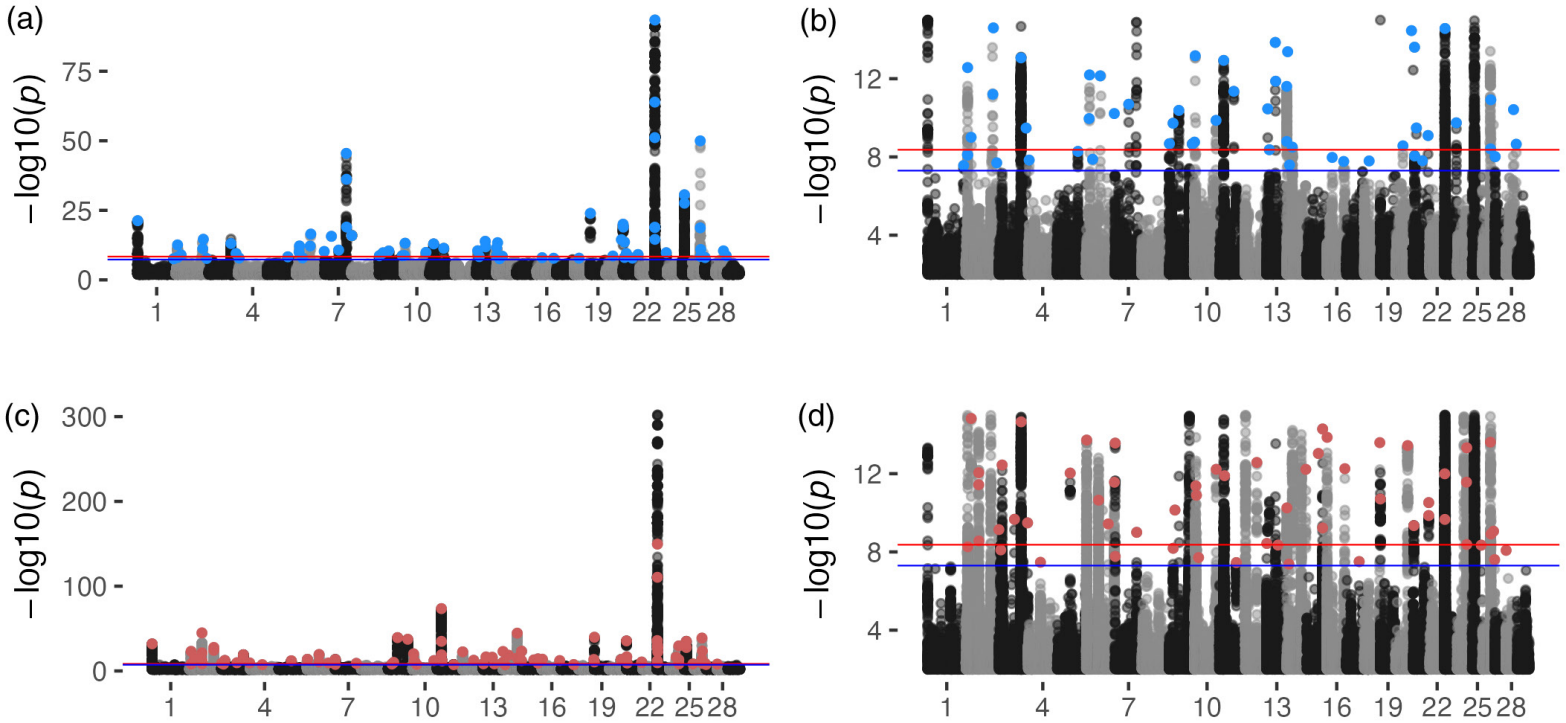
GPSM detects polygenic selection in the Red Angus dataset. Red Angus GPSM Manhattan plots for the (a) full dataset, (b) truncated at *p* = 10^−15^, and (c) young dataset also truncated (d) at *p* = 10^−5^. Genome‐wide significance is indicated by the blue line (*p*‐value = 5 × 10^−8^). Blue points are significant COJO SNPs (COJO *p* < 5 × 10^−5^) in the full Red Angus dataset. Red points are significant COJO SNPs in young Red Angus datasets.

GPSM identified 3617, 10,939, and 0 SNPs associated with birth date in the full, young, and old Red Angus datasets, respectively (*p* < 5 × 10^−8^) (Figure 1). The old dataset, which contained only 1984 individuals, was likely underpowered to detect signals of selection using GPSM. There were 3240 SNPs identified in the full dataset that were also significant in the young Red Angus data, a near‐complete overlap. The large proportion of new SNPs identified in the young Red Angus dataset was likely a function of increased power driving more nearby SNPs in LD with the selected allele above the significance threshold. Significant SNPs that were also in the 811 K marker set had an average imputation empirical *R* ^2^ value of 0.99. A stepwise conditional & joint analysis (COJO) (Yang et al., 2012) of these summary statistics further refined lead SNPs in significant loci (GPSM and COJO *p* < 5 × 10^−8^), which in many cases extended upwards of a megabase. This allowed us to pinpoint variants tightly linked to the causal mutations being selected in a computationally efficient manner. COJO identified 72 genome‐wide significant (*p* < 5 × 10^−8^) independent associations with birth date in the full dataset and 96 in the young dataset. Despite these datasets being largely composed of the same individuals and sharing a large proportion of common GPSM SNPs, only 16 SNPs identified by COJO were shared between both datasets.

Using COJO SNPs from the sequence‐level GPSM analysis, we annotated nearby genes, known quantitative trait loci (QTL), and other genomic features to help understand the biological pathways and phenotypes that selection targets in this population. In all datasets, the majority (51%–72%) of COJO SNPs resided within, or adjacent to (within 50 kb) annotated genes (Table 1). Depending on the dataset, 40% (full) or 54% (young) of these COJO SNPs with a clear positional candidate gene resided in regions immediately upstream or downstream of transcription start sites, insinuating that selection is primarily acting on *cis*‐regulatory regions of the genome. A complete accounting of nearby genes for COJO SNPs in the full and young Red Angus datasets is provided in Tables S5 and S6, respectively.

**TABLE 1:** Number of significant (COJO and genome‐wide *p* < 5 × 10^−8^) SNPs within, proximal, or outside of genic regions.

Breed	Dataset	Total COJO SNPs	Within gene	Gene proximal (<50 kb to nearest)	Intergenic
Red Angus	Full	72	17 (24%)	20 (28%)	35 (48%)
Red Angus	Young	96	36 (38%)	24 (25%)	36 (38%)
Simmental	Full	108	32 (30%)	36 (33%)	40 (37%)
Simmental	Purebred	18	6 (33%)	7 (39%)	3 (17%)

**FIGURE 2:**
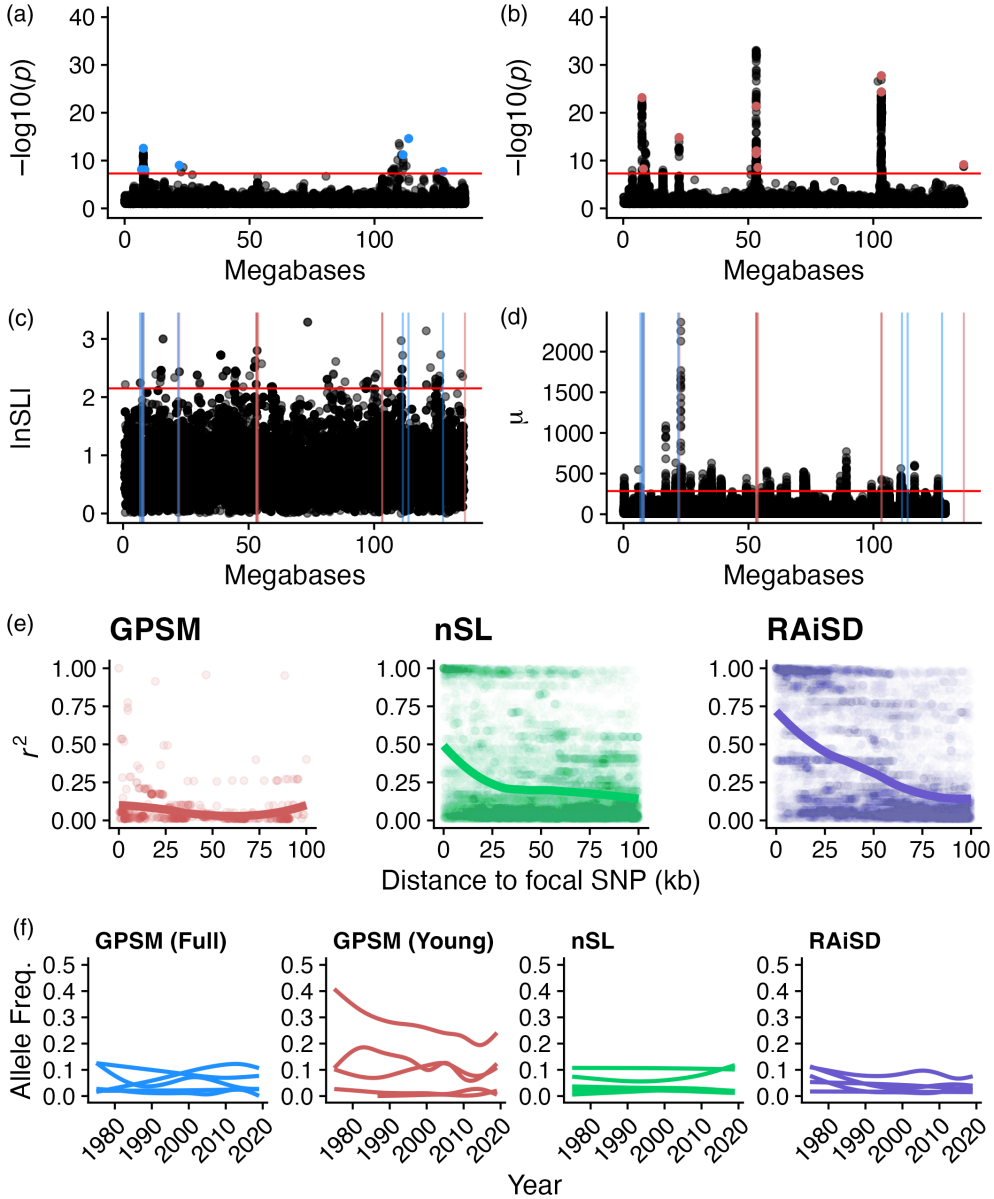
Methods identify largely different regions of selection on chromosome 2 in Red Angus. Chromosome 2 Manhattan plots for (a) full and (b) young Red Angus datasets. The blue line represents a genome‐wide significance threshold of 5 × 10^−8^. (c) Genomic distribution of |nSL| scores for windows defined by GenWin and (d) RAiSD μ statistics. Horizontal red lines indicate 0.05% outlier genome‐wide thresholds. Vertical red and blue lines represent the positions of full Red Angus and young Red Angus GPSM COJO SNPs, respectively. (e) Pairwise linkage disequilibrium (*r* ^2^) values for SNPs within 100 kb of COJO SNPs for GPSM, SNPs closest to the center of significant nSL windows, or lead SNPs in significant RAiSD peaks. (f) Allele frequency trajectories over time for the five most significant SNPs in each analysis.

In many cases, GPSM in young Red Angus animals detects novel signatures of recent ongoing selection that are not significant in the full dataset. Chromosome 2 offers an interesting case study of these differences. In addition to the two major peaks identified by both the full and young Red Angus datasets, at least eight additional major peaks are identified in the young data, accounting for 48 unique COJO associations (Figure 2a,b). The strongest unique associations identified in the young Red Angus dataset reside within the gene *ARHGAP15*. This association contains four unique COJO SNPs within the gene. *ARHGAP15* is a major gene involved in trypanotolerance in African cattle (Álvarez et al., 2016; Noyes et al., 2011), and likely has wider effects on immune function in worldwide cattle populations. Further, *ARHGAP15* is almost exclusively expressed in immune tissues (Fang et al., 2020). While some signatures represented sizeable allele frequency shifts, many were quite small (Figure 2f). As with any genome‐wide association study, SNP *p*‐values are a function of effect size and standard error, meaning that these genome‐wide significant SNPs could represent very small directional allele frequency shifts (effect estimates) with tiny standard errors. In other words, GPSM is picking up small, but consistent, changes in allele frequency due to selection.

Previously identified and annotated QTL for cattle allowed us to interpret the biological and production impact of selection decisions reflected in the GPSM results. By using QTL annotations, we can extrapolate the combined selective pressures on a population without having to ascribe a particular genomic feature to a selected locus (e.g., a mutation within a transcription factor binding site). GPSM signals overlapped with two well‐known QTL for metabolic body weight on chromosomes 14 (23.3 Mb) and 6 (38.5 Mb). Other QTL pointed toward ongoing selection for increased fertility and calving ease, known selection goals in the breed (https://redangus.org/about‐red‐angus/history/). We identified multiple GPSM COJO SNPs that overlap pleiotropic QTL mapped by Smith et al. (2022) using this same Red Angus study population. This includes multiple SNPs on chromosomes 14 (14:23257180:G:A) and 7 (7:88800495:C:T). Calving ease, sexual precocity, and carcass weight QTL were also identified among QTL tagged by GPSM COJO SNPs across the genome. No gene ontologies or pathways were enriched from the full Red Angus GPSM analysis. In the young Red Angus GPSM results, 12 terms were enriched (Table S9), mostly driven by three genes (ENSBTAG00000049666/*CYP3A5*, ENSBTAG00000052665/*CYP3A4*, ENSBTAG00000053645/*CYP3A5*).

### 3.4 Generation proxy selection mapping in Simmental

We analyzed the purebred Simmentals (by breed association definition, animals with greater than 87.5% Simmental pedigree ancestry) in our dataset to identify ongoing selection restricted to the breed. We performed an additional sequence‐level GPSM on the full Simmental dataset (all animals with ≥5% reported Simmental ancestry), representing selection within the entire herdbook, including the full spectrum of admixed animals (Figure S5).

**FIGURE 3:**
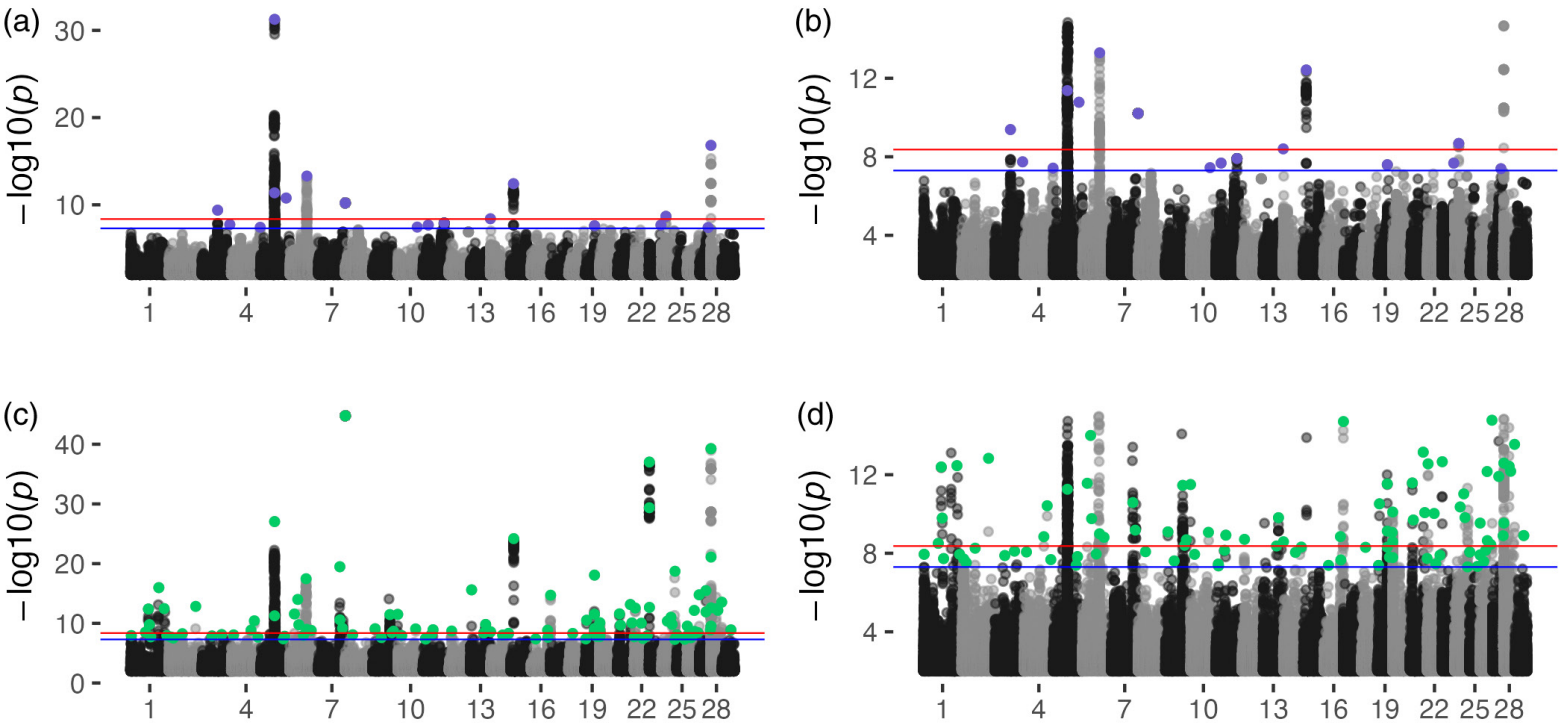
GPSM detects signatures of ongoing polygenic selection in the Simmental cattle. GPSM Manhattan plots for the (a) purebred Simmental dataset, (b) truncated at *p* = 10^−5^, and (c) full Simmental dataset also truncated (d) at *p* = 10^−5^. Genome‐wide significance is indicated by the blue line (*p*‐value = 5 × 10^−8^). Purple points indicate significant COJO SNPs (COJO *p* < 5 × 10^−5^) in the purebred Simmental dataset. Green points are significant COJO SNPs in the full Simmental dataset.

GPSM analysis of purebred Simmental animals (*n* = 13,379) identified 642 genome‐wide significant SNPs (Figure 3a,b) (*p* < 5 × 10^−8^). Significant loci were led by a strong signal on chromosome 5, centered at a locus containing *PMEL* and *ERBB3*, genes known to control coat color in Fleckvieh populations (i.e., European Simmental) (Mészáros et al., 2015). This locus, coupled with a GPSM signature immediately upstream of *KIT* (Durkin et al., 2012), suggest that the strongest selection pressures in the purebred American Simmental population have been on changing coat color and external appearance, making them appear less like European Simmental and more like American Angus. While these loci likely represent strong selection on monogenic or oligogenic traits, the subtle allele frequency shifts identified by GPSM elsewhere across the genome indicate that it is capable of detecting all types of directional allele frequency shifts. The next most significant locus resides in a cluster of olfactory receptors on chromosome 28. Another strong signal exists on chromosome 15 near beta‐carotene oxygenase 2 (*BCO2*) and interleukin‐18 (*IL‐18*), a locus likely involved in immune functions (He et al., 2010). Within these SNPs, COJO identified 18 independent associations, two of which were located at the center of chromosome 5 in the *PMEL/ERBB3* locus.

A GPSM analysis of all registered Simmental animals with at least 5% Simmental pedigree ancestry (*n* = 78,787) identified 1356 genome‐wide significant SNPs (*p* < 5 × 10^−8^) (Figure 3c,d). A COJO analysis found 108 independently associated SNPs (GPSM and COJO *p* < 5 × 10^−8^), two of which were identified in the purebred analysis (14:28004:G:T, 8:61247:T:C). These regions include the same significant coat color‐associated regions on chromosomes 5 and 6 and olfactory receptor clusters on chromosome 28 mentioned above. Two of the four major growth QTL that were identified in Braz et al. (2021) also appeared to be under selection in the full Simmental dataset. Three GPSM COJO SNPs resided in the chromosome 6 QTL (6:18953217:T:G, 6:32793962:G:A, 6:37125595:C:T), while two appeared in the QTL on chromosome 7 (7:87911770:A:C, 7:88579496:C:T). The major QTL on chromosome 6 likely represents ongoing selection at the *NCAPG‐LCORL* locus, a known driver of growth across mammalian species (Takasuga, 2016).

By subsetting these data, we identified recent Simmental‐specific selection in the purebred dataset and contrasted it with signatures identified in the full dataset where introgression from other breeds is also responsible for changing allele frequencies. In the full dataset, the most significant COJO SNP (9:85445646:T:C, COJO *p* = 2.51 × 10^−276^) is located immediately upstream of *SASH1*, a gene implicated in various fertility phenotypes in beef cattle (Fortes et al., 2010; Sweett et al., 2020; Xiang et al., 2017). Although not previously associated with pigmentation in cattle, SASH1 influences hair color and pigmentation in humans, both in Mendelian (OMIM, 2022; Zhang et al., 2017) and complex (Buniello et al., 2019; Kichaev et al., 2019) modes of inheritance. We also identified a strong selection of variants (4:94308044:A:G, COJO *p* = 2.17 × 10^−170^) in the imprinted gene *COPG2* (Khatib et al., 2007). As in Red Angus, the majority of GPSM COJO SNPs were either in or proximal to, (<50 kb from) genes (Table 1). 29% of the full and 33% of purebred COJO SNPs resided within genes. An additional 33% and 39% of COJO SNPs resided in close proximity to genes in the full and purebred datasets, respectively. A complete accounting of Simmental GPSM COJO detected SNPs and their nearby genes are in Tables S10 and S11.

In the purebred Simmental dataset, two of the most significant GPSM SNPs were near QTL involved with the appearance‐based traits of eye pigmentation and coat color on chromosomes 5 and 6 (Table S12). We also identified overlaps between GPSM loci and QTL for carcass (longissimus muscle area and carcass weight), production (metabolic body weight, average daily gain, dry matter intake), and reproduction (Inhibin level, luteal activity) traits (Table S13). For both Simmental datasets, gene sets related to keratinization were enriched (Tables S14 and S15).

### 3.5 GPSM identifies breed‐specific balancing selection in KHDRBS2 regulatory regions

**FIGURE 4:**
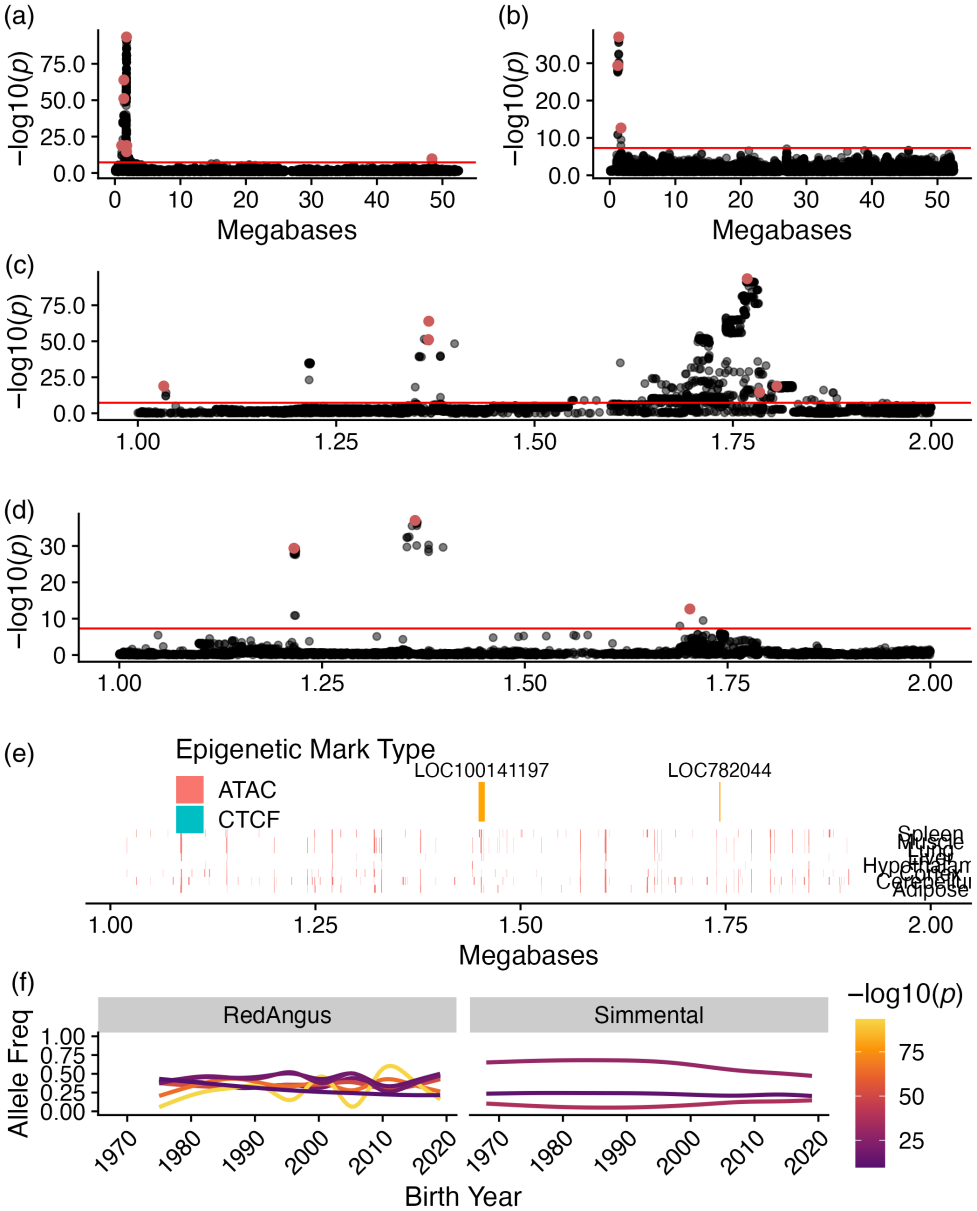
GPSM identifies a strong, complex signature of selection on chromosome 23. Manhattan plots for chromosome 23 in full (a) Red Angus and (b) Simmental datasets. Focused Manhattan plots at significant locus from 1 to 2 Mb on Chromosome 23 in full (c) Red Angus and (d) Simmental datasets. Red SNPs are significant GPSM COJO associations (COJO *p* < 5 × 10^−8^). In (a–d) the blueline indicates a genome‐wide significance (*p* < 5 × 10^−8^). (e) Chromosome 23 (1–2 Mb) annotated with genes (orange) and epigenetic marks in eight tissues from two bovine samples in the FAANG project, colored by mark type. (f) Allele frequency trajectories represented by smoothened regression lines of the birth year versus allele frequency for significant COJO SNPs in this region in the young Red Angus and full Simmental datasets. Lines are colored by the SNP's −log_10_(*p*) value from GPSM.

The most significant GPSM locus in both Red Angus datasets resided between 1 and 2 Mb on Chromosome 23 (Figure 4a,b). In this locus, 117 SNPs had *p*‐values < 10^−310^, reported by GCTA as zero in the young Red Angus dataset. This included the most significant SNP in the full dataset (23:1768070, *p* = 3.86 × 10^−94^) (Figure 4c). Interestingly, it was not the most significant SNP in the full Simmental dataset. Rather, a SNP ~550 kb away (23:1215338, COJO *p* = 4.98 × 10^−238^) had the strongest association (Figure 4d). Forty‐one SNPs within this locus were also significant in the full Simmental dataset (*p* < 5 × 10^−8^), including three independent COJO associations.

The closest protein‐coding gene to this selection signature is *KHDRBS2*, a gene involved in reproduction in goats (Islam et al., 2020), and calving ease in cattle (Cole et al., 2011). *KHDRBS2* has also been identified as possessing a selection signature that differs between *Bos taurus* and *Bos indicus* cattle (Paim et al., 2020; Pérez O'Brien et al., 2014). While our most significant SNPs did not reside directly in known epigenetic mark regions (Figure 4e), dozens of marks exist within this 1 Mb segment. Further, the annotated and expressed pseudogene *LOC782044* is 24,991 base pairs from the most significant GPSM association in Red Angus at 1,768,070 base pairs and 38,847 base pairs from a significant COJO SNP in Simmental. While *LOC782044* does not have any known functions in cattle, it could be an enhancer RNA (a common hallmark on long noncoding RNAs) transcribed from an enhancer altering the expression of *KHDRBS2* or other nearby genes (Sartorelli & Lauberth, 2020).

The observed allele frequencies at significant SNPs in this locus show two major patterns. First, we observe small directional changes in frequency, consistent with the polygenic selection that we observe at other GPSM loci (Figure 4f). Second, for some lead SNPs in Red Angus, we observe allele frequencies oscillating around an intermediate value, a pattern consistent with balancing selection (Orozco‐terWengel et al., 2012). These fluctuations over very short time periods appear as directional, but additional evidence points toward ongoing balancing selection. This region is enriched for high Tajima's *D* scores, where 25% of windows have a Tajima's *D* score > 2 (top 10%), indicative of higher‐than‐expected levels of genetic variation. The highest Tajima's *D* score genome‐wide was located in a window ~70 kb from the most significant GPSM SNPs.

### 3.6 Sweep mapping identifies known and novel selected loci

**FIGURE 5:**
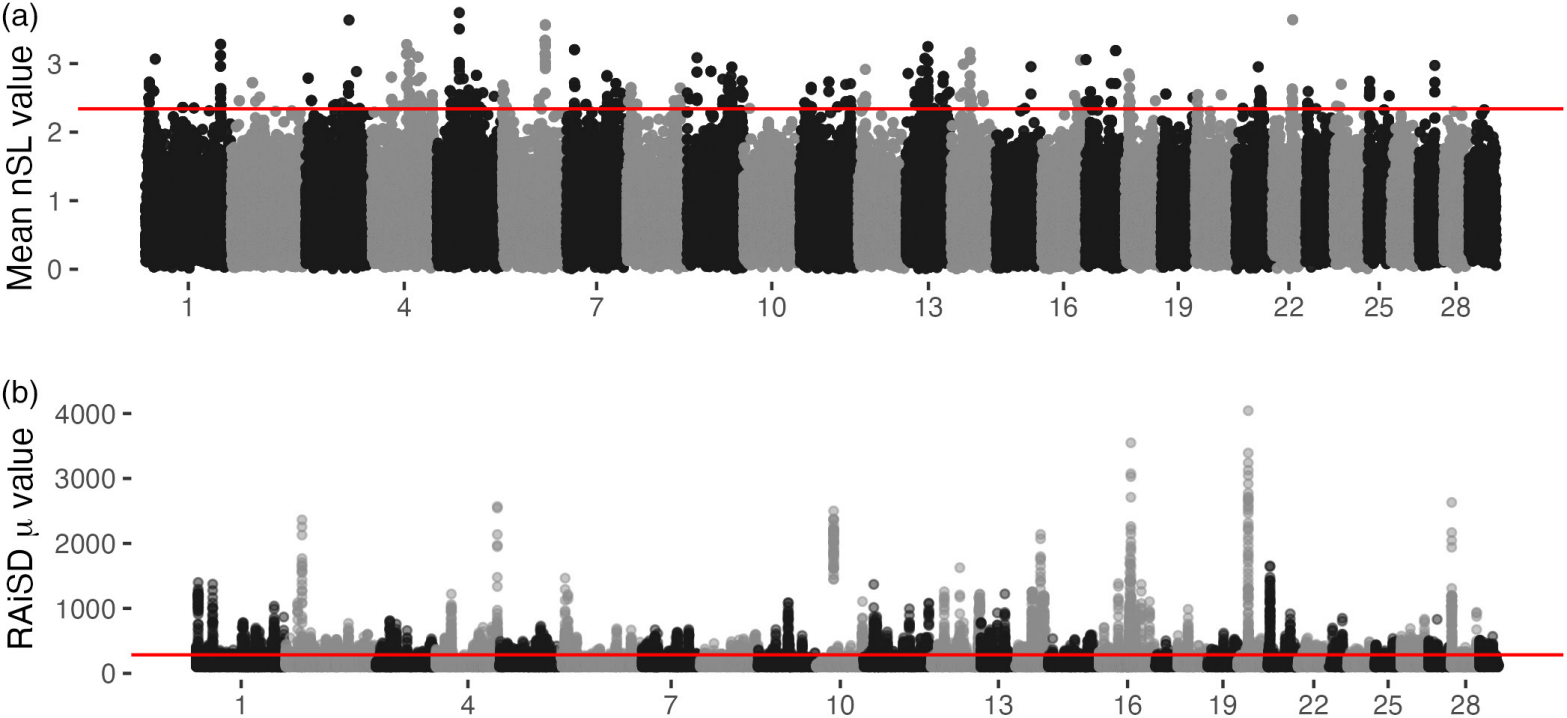
Selective sweep mapping in Red Angus. (a) Number of segregating loci (nSL) statistic windows across the genome. Window boundaries were defined by GenWin R package. Points represent the average |nSL| values within a window, with the genomic position defined as the genomic center of the window. Red line delineates 0.5% outliers deemed significant. (b) Manhattan plot of RAiSD μ statistics calculated from sequenced American Red Angus animals in the 1000 Bull Genomes Project. Red line delineates 0.05% outliers.

GPSM and traditional selective sweep methods are both focused on identifying allele frequency changes and/or the signatures that they leave on surrounding neutral sites. We used two selection mapping methods, a haplotype‐based statistic, number of segregating loci (nSL) (Ferrer‐Admetlla et al., 2014), and a composite statistic (μ) implemented by the software RAiSD (Alachiotis & Pavlidis, 2018). For nSL, we defined windows using the GenWin R package (Beissinger et al., 2015) which uses a spline function to observe changes in test statistics, and called the top 0.5% of windows significant, provided they contained at least three SNPs. Our nSL analysis in Red Angus identified 365 outlier windows on all but three chromosomes (17, 27, and 28) (Table S16, Figure 5). The correlation between nSL scores and GPSM effect sizes for 811 K SNPs was −0.007. None of the significant GPSM COJO SNPs resided in outlier nSL windows. We also used RAiSD to look for site frequency spectrum differences indicative of selection. RAiSD calculates μ in 50 SNP sliding windows (mean length = 39,139 bp), and we considered windows with the top 0.05% of μ values as signatures of selection (Table S17). The RAiSD analysis of sequenced Red Angus animals in the Thousand Bulls Project (*n* = 14) (Hayes & Daetwyler, 2019) identified 3740 significant windows, many of which were overlapping, that encompassed at least 339 loci exhibiting sweep‐like signatures.

Of the 3740 significant windows identified by RAiSD, 17 showed an overlap with three distinct COJO associations in the young Red Angus dataset, but we did not observe any overlap with COJO SNPs in the full Red Angus dataset. In Red Angus, there were 12 sweep regions identified by both RAiSD and nSL. These included a signature on chromosome 6 (~78.95 Mb) that resides in a pleiotropic QTL for stayability, calving ease, and udder structure in dairy cattle, traits all under selection in the Red Angus breed (Cole et al., 2011). Another shared sweep region on chromosome 11 (18.1 Mb) is associated with multiple carcass quality traits (Mateescu et al., 2017).

While the associated regions were largely different from those identified by GPSM analysis, we identified 12 GPSM COJO SNPs (4 in full and 8 in young Red Angus datasets, respectively) that reside within 50 kb of a significant nSL window. Two of these loci reside on chromosome 14 (23.0 and 58.1 Mb). The locus at 23.0 Mb was also identified as a significant sweep region by RAiSD. This locus resides within the gene *TMEM68* which has previously been identified as a driver of feed intake and growth phenotypes in cattle (Lindholm‐Perry et al., 2012), and height in humans (Kichaev et al., 2019). This locus also resides within a QTL for Insulin‐like growth factor 1 level (Fortes et al., 2013). The locus at 58.1 Mb lies within the gene Oxidation Resistance 1 (*OXR1*), which is also a known regulator of carcass weight in cattle (Zhang et al., 2019) and neutrophil counts in humans (Chen et al., 2020). A final shared region on chromosome 12 includes a region located immediately downstream of the gene *DNAJC15*, a heat shock protein with multiple reproductive associations in cattle (Cochran et al., 2013; Zhang et al., 2011) and human birth weight (Comuzzie et al., 2012).

We expect that selective sweeps assert different pressures on neighboring neutral sites compared with polygenic selection. To quantify these differences, we calculated linkage disequilibrium *r* ^2^ statistics for all imputed SNPs within 100 kb of the lead SNP in significant RAiSD (*n* = 35) and nSL (*n* = 40) windows and significant GPSM COJO SNPs (*n* = 7) on chromosome 2 (Figure 2e). On average the *r* ^2^ in regions surrounding sweep loci (RAiSD loci mean *r* ^2^ = 0.223 [SD = 0.324], nSL loci mean *r* ^2^ = 0.155 [SD = 0.205]) were significantly higher (Tukey HSD *p*‐value < 2 × 10^−16^) than those around GPSM loci (mean *r* ^2^ = 0.092 [SD = 0.192]). Unlike GPSM results, across the four sweep analyses, at minimum 47% of identified windows overlapped genes (Table 2).

**TABLE 2:** Number of significant windows within, proximal, or outside of genic regions identified by nSL and RAiSD.

Breed	Analysis	Total windows	Within gene	Gene proximal (<50 kb to nearest)	Intergenic
Red Angus	nSL	365	183 (50%)	73 (20%)	109 (30%)
Red Angus	RAiSD	6502	3453 (53%)	811 (13%)	2238 (34%)
Simmental	nSL	347	188 (54%)	60 (17%)	99 (29%)
Simmental	RAiSD	6497	3030 (47%)	1108 (17%)	2359 (36%)

**FIGURE 6:**
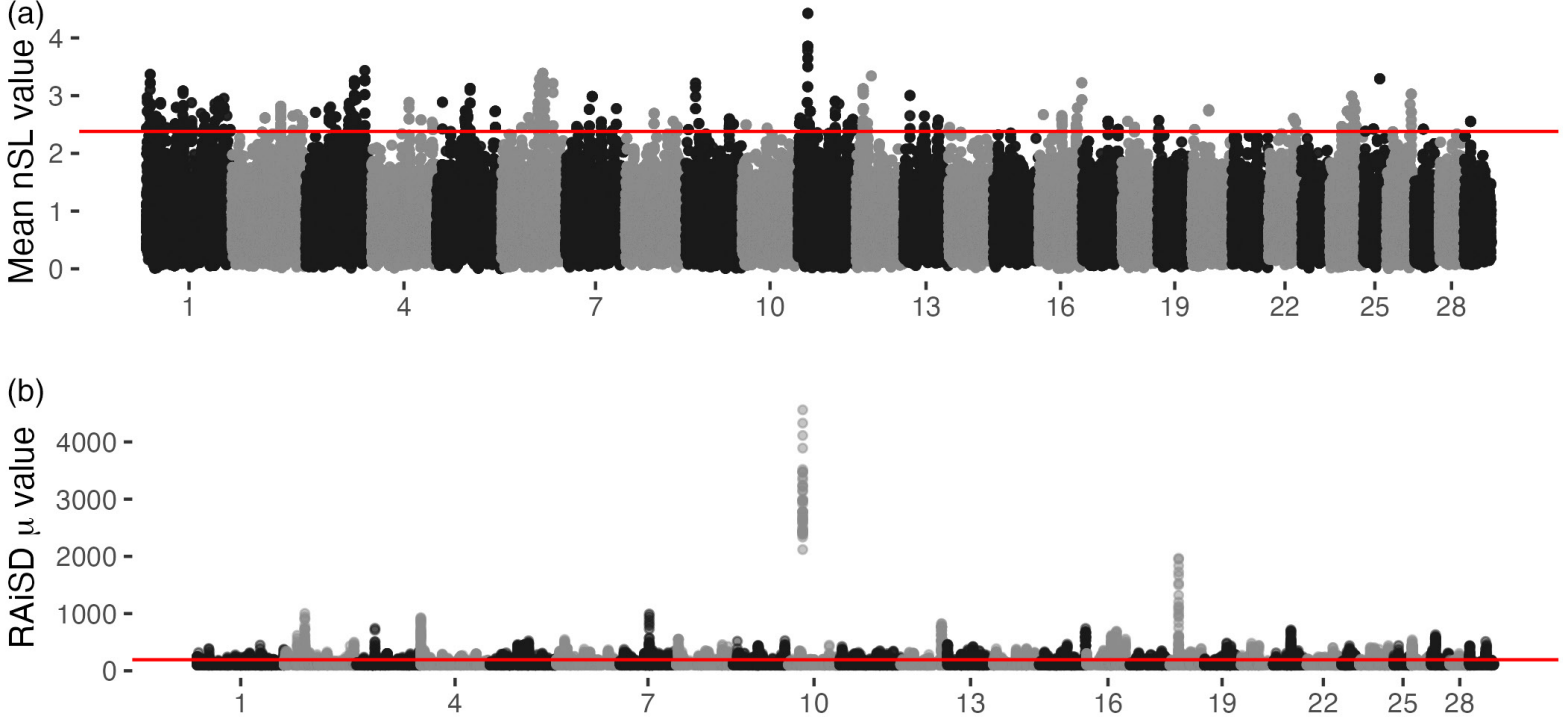
Selective sweep mapping in Simmental. (a) Number of segregating loci (nSL) statistic windows across the genome. Window boundaries were defined by the GenWin R package. Points represent the average |nSL| values within a window, with the genomic position defined as the genomic center of the window. Red line delineates 0.5% outliers deemed significant. (b) Manhattan plot of RAiSD μ statistic calculated from sequenced American Simmental animals in the 1000 Bull Genomes Project. Red line delineates 0.05% outliers.

Similarly, low levels of overlap between GPSM and sweep detection methods existed in Simmental (Figure 6). A single GPSM COJO SNP resided within a significant RAiSD window (chromosome 26 at 637,478 base pairs). The lone overlap between nSL and GPSM COJO SNPs in Simmental was on chromosome 5 at 57,701,350 base pairs. This is approximately 500 kb from the *PMEL*/*ERBB3* coat color candidate locus and resides within a cluster of olfactory‐associated genes. RAiSD and nSL analyses in Simmental identified at least 14 shared regions of selection (significant windows <50 kb apart). This complementary evidence increases our confidence that an actual sweep occurred at a locus. The most notable shared locus between nSL and RAiSD is located at the *POLLED* locus (Medugorac et al., 2012; Wiedemar et al., 2014) on chromosome 1, responsible for the presence or absence of horns. This locus has been under strong selection in most cattle populations and is frequently identified in selection mapping studies (Kemper et al., 2014; Xu et al., 2015). Another shared region of selection on chromosome 16 at ~42.6 Mb near the genes *MASP2* and *TARDBP* has been identified in numerous other sweep mapping studies of Simmental cattle (Qanbari et al., 2014; Ramey et al., 2013; Rothammer et al., 2013; Zhao et al., 2015).

While overlapping nSL and RAiSD signatures can help bolster confidence in candidate selective sweeps, most windows are uniquely identified by a single method (Tables S16–S19). For example, the locus with the largest RAiSD μ statistic (Figures 5 and 6) is located between two major clusters of T cell receptors on chromosome 10 at ~24.2 Mb. This sweep region was also identified in European Simmental populations by Qanbari et al. (2014) and Zhao et al. (2015), illustrating the long‐lasting signatures created by some selective sweeps. The next highest RAiSD μ statistic windows in Simmental are located immediately upstream of *MC1R*, the gene responsible for red versus black coat color in cattle (Gutiérrez‐Gil et al., 2007). The most significant nSL signature with a clear candidate gene resided within *TMEM132D*, a gene that has been identified in numerous selective sweep analyses (Mészáros et al., 2019; Moradian et al., 2020; Moscarelli et al., 2020; Qanbari et al., 2014) and is pleiotropic across dairy and beef cattle breeds (Martins et al., 2020).

### 3.7 Sweep and polygenic selection mapping identify selection on similar complex traits

Using regions identified by RAiSD and nSL, we performed gene set enrichment analyses to identify the biological components that underlie sweep regions in contemporary cattle populations. Gene set enrichment analyses for nSL analyses of Red Angus did not identify any significant ontologies or pathways. Metabolic pathways were the only enriched KEGG term (Kanehisa & Goto, 2000) from the Simmental nSL gene set (Table S20). Despite the lack of overlapping enrichments between nSL regions in Red Angus and Simmental, results from RAiSD gene enrichment analyses in the two populations identified multiple shared ontologies involved in development and binding (Tables S21 and S22). These shared ontologies included embryonic skeletal system development and morphogenesis, and biological functions likely to be under selection during breed formation and improvement. Multiple additional terms involved in development and morphogenesis were enriched in the Red Angus RAiSD gene set. Terms enriched uniquely in Simmental tended to be involved more with cytoskeletal binding (microtubule binding, protein binding, tubulin binding, etc.).

We used cataloged bovine QTL to identify sweep regions that are also associated with complex traits. Enrichment analyses for each breed and detection method identified numerous QTL classes that were significantly overrepresented in sweep regions. Despite the minimal overlap between loci, most GPSM‐enriched QTL classes were also enriched in nSL or RAiSD regions. In Red Angus, four of the five QTL classes enriched in GPSM loci were also enriched in sweep regions (metabolic body weight – nSL, calving ease – RAiSD, sexual precocity – RAiSD, and carcass weight – both) (Tables S23 and S24). All five QTL classes identified near GPSM COJO SNPs in Simmental (longissimus muscle area, average daily gain, dry matter intake, metabolic body weight, and carcass weight) were also significantly enriched in significant nSL regions (Table S25). In many cases, these enrichments were driven by QTL located on a single chromosome.

The most significantly enriched QTL classes in Red Angus were calving ease (RAiSD QTL enrichment *p*‐value = 1.00 × 10^−109^) and luteal activity (nSL QTL enrichment *p*‐value = 2.73 × 10^−64^). Luteal activity was also the most significant QTL class identified in the Simmental nSL regions (enrichment *p*‐value = 1.39 × 10^−120^). In each case, the luteal phase QTL class was enriched due to a single signature on chromosome 3 that encompassed 163 annotated QTL. QTL for eye pigmentation area and facial pigmentation were also enriched in Simmental nSL regions driven exclusively by a region on chromosome 6 that was also identified by GPSM.

## 4 DISCUSSION

In this study, we further demonstrate the power of linear mixed models applied to a novel dependent variable for detecting ongoing selection in populations with temporal genotype data. The Generation Proxy Selection Mapping (GPSM) method (Decker et al., 2012; Rowan et al., 2021) is unique among selection mapping methods because it does not rely on outlier definitions and significance is calculated on a per‐marker basis, allowing us to pinpoint selection to very small intervals. Building on the work of Rowan et al. (2021), we expand GPSM to significantly larger datasets (46,454 and 78,787 animals) with imputed sequence‐level variants (>11 million SNPs) (Rowan et al., 2021). Imputation is accurate both from low‐density to high‐density chip and from high‐density to sequence‐density due to our large reference datasets and the large LD blocks in cattle populations. A large‐scale validation using large multi‐breed reference panels to impute from low‐density chips to 811 K SNPs resulted in per‐animal imputation accuracies greater than 0.99 for both Simmental and Red Angus (Rowan et al., 2019). By restricting sequence‐imputed data to those with estimated *R* ^2^ values >0.4, we avoid performing association tests on variants likely to be imputed incorrectly. Empirical *R* ^2^ values for genotyped variants were highly accurate (>0.99 in both Red Angus and Simmental). Further, we would expect that in cases where high uncertainty in imputation exists, the GPSM would experience a decrease in power, as genotypes would be more similar across generations, not causing false positives. This boost in power and resolution from imputation allowed us to map hundreds of small directional shifts in allele frequency, consistent with polygenic selection (Rosenberg et al., 2019). Further, using a genomic relationship matrix, we are better able to control for the demography of the populations.

Due to the non‐normality of generation proxy phenotypes in genotyped livestock populations, we explored the effects of variable transformation and data subsetting on GPSM's ability to detect selection. Large residuals for individuals born in the distant past led to a reduced power to detect selection in the full Red Angus dataset. When subsetting this dataset to individuals born very recently, or Box‐Cox transforming the “birth date” generation proxy, we detected three times as many birth date‐associated SNPs. In populations with low effective sizes (N_e_), we might expect that stochastic changes in allele frequency due to drift could generate detectable changes in frequency (Nei & Tajima, 1981), but simulations performed in our previous work have shown that GPSM is effectively able to distinguish between drift and selection (Rowan et al., 2021). Simulations also suggested that non‐normal distributions of phenotypes led to reduced power to detect selection over equivalent time periods (Rowan et al., 2021). While a Box‐Cox‐transformed birth date generation proxy boosted the significance of signals in Red Angus, a similar optimal power transformation did not have the same effect when applied to Simmental animals. As a result, for sequence‐level analyses, we used the untransformed birth date as our dependent variable but partitioned the data into subsets to probe different components of selection.

While most other tests of polygenic selection explicitly test for correlations between allele frequencies and phenotypes over sampled time periods (Beissinger et al., 2018; Szpiech et al., 2020; Turchin et al., 2012), or between distinct diverged populations (Chen et al., 2010; Szpiech et al., 2020), GPSM operates agnostic to phenotype and population label. This expands the odds of detecting polygenic selection signatures but can make interpreting signals difficult. Fortunately, hundreds of QTL‐mapping studies have been performed in cattle, providing an extensive database of loci known to affect economically important complex traits (Hu et al., 2019). We queried the Animal QTL Database for QTL near GPSM associations and performed enrichment tests to understand the traits under selection in these populations. In Red Angus, we identified significant QTL enrichments for several production traits such as body weight, average daily gain, and carcass weight, all traits that we would expect to find under selection in beef populations. We also identified multiple QTL classes influencing maternal traits and calving ease, two major recent selection emphases in the Red Angus breed. By analyzing both the full Simmental dataset and a subset of purebred animals, we could disentangle which allele frequencies were changing due to Simmental‐specific selection versus selection on variation introgressed from other breeds. In purebred Simmental, we found a limited selection of traits that were not explicitly involved in appearance characteristics. Strong selection at the *PMEL*/*ERBB3* and *KIT* loci have been a major focus of the breed as it aimed at making animals appear more like Angus cattle. Along these lines, although not previously identified, *SASH1* may also be under selection in Simmental related to hair color. As a result, less selection on complex production traits was detected by GPSM in the purebred dataset compared with the full dataset where we detected an excess of associations with carcass, production, and reproductive traits, consistent with ongoing selection in the cattle industry at large. Differences in the enriched carcass and production traits are consistent with traits that show appreciable average phenotypic differences between Angus and Simmental animals. These QTL databases are far from comprehensive due to their biases toward loci with detectable effect sizes in frequently measured traits. In many cases, these enrichments were driven by what is likely a single QTL represented by multiple entries in the database. While this is useful for pointing out likely functions for a single locus, it may overestimate enrichments. As a result, for all QTL enrichments, we report the maximum number of QTL identified on any single chromosome and that chromosome. Regardless, they provide a valuable first step to categorizing the production traits driving genomic changes in these populations. Further, we anticipate that GPSM, nSL, and RAiSD loci will serve as valuable functional annotations for the bovine genome. As studies continue to map associations with complex traits, knowledge of the ongoing selective forces acting upon those loci will add valuable context and information.

While some overlap between selective sweep mapping outlier windows (nSL and RAiSD) and GPSM hits existed, they largely identified different genomic loci. An intuitive explanation of this result is that selective sweeps remove variation from the locus, thus there is little variation at that locus on which polygenic selection can act. Sweep mapping methods consistently identify important Mendelian loci, such as *POLLED*, coat color genes (*MC1R*, *KIT*, etc.), and large‐effect QTL where selection has for all intents and purposes been completed. GPSM's strength is in detecting subtle, directional shifts in allele frequency over short periods of time where selection is ongoing. In contrast, nSL, RAiSD, and other sweep mapping methods identify characteristics of sweeps such as extended haplotype homozygosity, long‐range LD, and changes to the local site frequency spectrum. We chose to use these two methods to represent methods aimed at detecting both hard (RAiSD) and soft (nSL) sweeps. We observed significantly less LD between GPSM SNPs and neighboring neutral sites compared with the lead SNPs in sweep peaks identified by nSL and RAiSD. In general, most sweep mapping strategies search for signatures at neighboring neutral sites, whereas GPSM tracks actual allele frequency changes over time. Sweep methods may have limited application in finding informative SNPs for genomic predictions, whereas GPSM is identifying the current targets of polygenic selection and should be highly informative.

In Red Angus and Simmental, we have identified the historical sweeps and ongoing selection that have altered the genotypes and phenotypes of these populations. By assessing the selection architecture (combination of polygenic selection, soft sweeps, and hard sweeps), we were better able to describe the history of selection in these two breeds. Loci identified by RAiSD μ statistic have altered linkage disequilibrium, sequence diversity, and site frequency spectrum indicative of severe sweeps. In both Red Angus and Simmental, the genes identified by RAiSD were enriched for developmental processes, such as “anatomical structure development” and “embryonic skeletal system morphogenesis”. This likely reflects historical visual selection for “type” (body shape) and breed character (how closely an animal matches the breed's ideal) that occurred prior to systematic data collection and mixed model genetic evaluations. For example, *HOXB1* was in the center of two selective sweeps identified by RAiSD in both Red Angus and Simmental. In human datasets, HOXB1 has been associated with waist circumference adjusted for body mass index (Zhu et al., 2020) and height (Yengo et al., 2022). Conversely, no functions were consistently enriched based on genes within nSL haplotype homozygosity regions, whose signatures reflect weaker, incomplete sweeps. Enriched QTL classes for nSL and RAiSD loci often highlight similar traits as GPSM loci, but for nSL and RAiSD loci these enriched QTL classes are often from multiple associations with a single chromosome.

Genes identified by GPSM reflect genetic and genomic selection for economically important traits. This agrees with previous reports, but with greater power and precision in the current study (Rowan et al., 2021). Genetic improvement in Red Angus is driven by selection within the breed, whereas improvement in Simmental appears to be driven by preferential introgression of certain Angus sires. Our results provide a detailed description of the selection architectures in two of the most common cattle breeds in America, reflecting a history of visual selection for breed type followed by a transition to modern genomic selection.

## 5 CONCLUSIONS

Using large, commercially generated cattle genotype datasets imputed to 11 million SNPs and the Generation Proxy Selection Mapping method we fine‐mapped hundreds of loci undergoing subtle directional shifts in frequency. These loci reside overwhelmingly in, or nearby, genes, which suggests that selection on complex traits is likely concerned with perturbing gene expression patterns in complex networks. GPSM detected largely different sets of selected loci than the selective sweep mapping methods. When longitudinally‐sampled genotypes are available, GPSM is a powerful method for detecting ongoing changes to the genome. This makes it a complementary approach to sweep mapping strategies as we work to understand the impacts of all types of selection on the genome.

## CONFLICT OF INTEREST STATEMENT

The authors declare no conflicts of interest related to this work.

## Supporting information


Appendix S1:

## Data Availability

The raw data underlying the results presented in the study are available from the Red Angus Association of America (Lindsay Upperman, Director of Breed Improvement, lindsay@redangus.org) or the American Simmental Association (Jackie Atkins, Director Science and Education Operations, jatkins@simmgene.com,) under a Data Use Agreement, but are not publicly available. Summary statistics for all analyses presented here are freely available as supplementary files.
