# Supplementary material for: Uncovering the architecture of selection in two Bos taurus cattle breeds
Source: Evol Appl. 2024 Feb 22;17(2):e13666. doi: 10.1111/eva.13666 (PMC10883790; doi:10.1111/eva.13666)
Supplement: Supplementary file 1 — Appendix S1: [file EVA-17-e13666-s001.zip › EA_SupplementaryFile1.docx]

**Supplementary Table 1**. Genomic restricted maximum likelihood (GREML) estimates of proportion variance explained (PVE) for various statistical transformations to birth date used as generation proxy in Red Angus.

| **Subset** | **n(animals)** | **Dependent Variable** | **PVE (SE)** |
| --- | --- | --- | --- |
| FULL | 46,454 | Birth date | 0.523 (0.007) |
| FULL | 46,456 | Birth date*^-0.237^* | 0.657 (0.006) |
| Young Only^1^ | 44,470 | Birth date | 0.773 (0.004) |
| Old Only^2^ | 1,984 | Birth date | 0.557 (0.031) |

^1^ Animals born on or after January 1, 2012

^2^ Animals born prior to January 1, 2012

**Supplementary Table 2**. Genomic restricted maximum likelihood (GREML) estimates of proportion variance explained (PVE) for various subsets of the Simmental dataset.

| **Dataset** | **n (animals)** | **Dependent Variable** | **PVE (Standard Error)** |
| --- | --- | --- | --- |
| Full^1^ | 78,787 | Birth date | 0.619 (0.005) |
| Full^1^ | 78,787 | Birth date^0.0345^ | 0.605 (0.005) |
| Young Only^2^ | 73,811 | Birth date | 0.540 (0.005) |
| Old Only^3^ | 4,976 | Birth date | 0.436 (0.021) |
| <30% SIM, >50% AN^4^ | 11,429 | Birth date | 0.665 (0.011) |
| >20% SIM, <70%SIM^5^ | 46,136 | Birth date | 0.642 (0.006) |
| >70% SIM^6^ | 31,225 | Birth date | 0.558 (0.008) |
| Purebred^7^ | 13,379 | Birth date | 0.555 (0.011) |
| Purebred Young^8^ | 11,148, | Birth date | 0.497 (0.013) |
| Purebred Old^9^ | 2,231 | Birth date | 0.462 (0.030) |

^1^ Animals with at least 5% Simmental ancestry

^2^ Animals with at least 5% Simmental ancestry, born on or after January 1, 2008

^3^ Animals with at least 5% Simmental ancestry, born prior to January 1, 2008

^4^ Animals with less than 30% Simmental ancestry and more than 50% Angus ancestry

^5^ Animals with more than 20%, but less than 70% Simmental ancestry

^6^ Animals with more than 70% Simmental ancestry

^7^ Animals with 100% Simmental ancestry

^8^ Animals with 100% Simmental ancestry, born on or after January 1, 2008

^9^ Animals with 100% Simmental ancestry, born prior to January 1, 2008

**Supplementary Table 3.** Counts of significant SNPs identified in each GPSM analysis of the Red Angus population using different transformations to generation proxy as dependent variable in 811K SNPs. The four significance cutoffs are reported: 1) A nominal significance (p < 10^-5^), 2) a Bonferroni-adjusted threshold (p < 7.55 x 10^-7^), and FDR-corrected q-values 3) < 0.10 or 4) < 0.05.

| **Dataset** | **n (animals)** | **Dependent Variable** | **nSNPs**  **p < 10^-5^ (nloci)** | **nSNPs p < 7.55 x 10^-7^ (nloci)** | **nSNPs q < 0.10 (nloci)** | **nSNPs q < 0.05**  **(nloci)** |
| --- | --- | --- | --- | --- | --- | --- |
| Full | 46,454 | Birth date | 315 | 214 | 509 | 398 |
| Full | 46,454 | Birth date^-0.237^ | 540 | 390 | 907 | 754 |
| Young Only^1^ | 44,470 | Birth date | 762 | 555 | 1210 | 1042 |
| Old Only^2^ | 1,984 | Birth date | 18 | 1 | 1 | 1 |

^1^ Animals born on or after January 1, 2012

^2^ Animals born prior to January 1, 2012

**Supplementary Table 4**. Counts of significant SNPs identified in each GPSM analysis of the Simmental population using different population subsets and transformations to generation proxy with 811K SNPs. Ancestry proportions are pedigree estimates reported by the American Simmental Association. The four significance thresholds are reported: 1) A nominal significance (p < 10^-5^), 2) a Bonferroni-adjusted threshold (p < 7.55 x 10^-7^), and FDR-corrected q-values 3) < 0.10 or 4) < 0.05.

| **Dataset** | **n (animals)** | **Dependent Variable** | **Nominal  (p < 10^-5^)** | **Bonferroni** | **q < 0.1** | **q < 0.05** |
| --- | --- | --- | --- | --- | --- | --- |
| Full^1^ | 78,787 | Birth date | 120 | 70 | 137 | 117 |
| Full^1^ | 78,787 | Birth date^0.0345^ | 109 | 77 | 130 | 105 |
| Young Only^2^ | 73,811 | Birth date | 94 | 68 | 100 | 89 |
| Old Only^3^ | 4,976 | Birth date | 119 | 72 | 171 | 115 |
| <30% SIM, >50% AN^4^ | 11,429 | Birth date | 14 | 3 | 3 | 0 |
| >20% SIM, <70%SIM^5^ | 46,136 | Birth date | 46 | 31 | 39 | 38 |
| >70% SIM^6^ | 31,225 | Birth date | 100 | 61 | 107 | 88 |
| >70% SIM^6^ | 31,225 | $log(Birth date)$ | 51 | 24 | 44 | 32 |
| Purebred^7^ | 13,379 | Birth date | 92 | 50 | 111 | 85 |
| Purebred Young^8^ | 11,148, | Birth date | 11 | 4 | 4 | 3 |
| Purebred Old^9^ | 2,231 | Birth date | 60 | 34 | 59 | 48 |

^1^ Animals with at least 5% Simmental ancestry

^2^ Animals with at least 5% Simmental ancestry, born on or after January 1, 2008

^3^ Animals with at least 5% Simmental ancestry, born prior to January 1, 2008

^4^ Animals with less than 30% Simmental ancestry and more than 50% Angus ancestry

^5^ Animals with more than 20%, but less than 70% Simmental ancestry

^6^ Animals with more than 70% Simmental ancestry

^7^ Animals with 100% Simmental ancestry

^8^ Animals with 100% Simmental ancestry, born on or after January 1, 2008

^9^ Animals with 100% Simmental ancestry, born prior to January 1, 2008

**Supplementary Tables 5-26 are in Supplementary File 2.**

**Supplementary Table 5.** Significant (p < 5 x 10^-8^) conditional and joint (COJO) SNPs from full Red Angus GPSM analysis and their annotated nearby genes (< 50 kb).

**Supplementary Table 6.** Significant (p < 5 x 10^-8^) conditional and joint (COJO) SNPs from young Red Angus GPSM analysis and their annotated nearby genes (< 50 kb).

**Supplementary Table 7.** Significantly enriched QTL in regions (< 50 kb) from significant GPSM COJO SNPs identified in the full Red Angus dataset (n SNPs = 248) .

**Supplementary Table 8.** Significantly enriched QTL in regions (< 50 kb) from significant GPSM COJO SNPs identified in the young Red Angus dataset (n SNPs = 417).

**Supplementary Table 9**. Significantly enriched gene set annotations in regions (< 50 kb) from significant GPSM COJO SNPs identified in the young Red Angus dataset (n SNPs = 417).

**Supplementary Table 10.** Significant (p < 5 x 10^-8^) conditional and joint (COJO) SNPs from full Simmental GPSM analysis and their annotated nearby genes (< 50 kb).

**Supplementary Table 11.** Significant (p < 5 x 10^-8^) conditional and joint (COJO) SNPs from purebred Simmental GPSM analysis and their annotated nearby genes (< 50 kb).

**Supplementary Table 12.** Significantly enriched QTL in regions (< 50 kb) from significant GPSM COJO SNPs identified in full Simmental dataset (n SNPs = 344).

**Supplementary Table 13.** Significantly enriched QTL in regions (< 50 kb) from significant GPSM COJO SNPs identified in purebred Simmental dataset (n SNPs = 33).

**Supplementary Table 14**. Significantly enriched gene set annotations in regions (< 50 kb) from significant GPSM COJO SNPs identified in the full Simmental dataset (n SNPs = 344).

**Supplementary Table 15.** Significantly enriched gene set annotations in regions (< 50 kb) from significant GPSM COJO SNPs identified in the purebred Simmental dataset (n SNPs = 33).

**Supplementary Table 16.** Red Angus outlier nSL windows (top 0.5%) as defined by GenWin R package. Genes that fell within significant windows are reported with their corresponding window.

**Supplementary Table 17.** Unique genes within Red Angus outlier RAiSD windows (top 0.05%). Reported window is the window with the highest μ value that overlaps with the gene.

**Supplementary Table 18.** Simmental outlier nSL windows (top 0.5%) as defined by GenWin R package. Genes that fell within significant windows are reported with their corresponding window.

**Supplementary Table 19.** Unique genes within Simmental outlier RAiSD windows (top 0.05%). Reported window is the window with the highest μ value that overlaps with the gene.

**Supplementary Table 20.** Significantly enriched gene set annotations of genes within Simmental outlier nSL windows.

**Supplementary Table 21.** Significantly enriched gene set annotations of genes within Red Angus outlier RAiSD windows.

**Supplementary Table 22.** Significantly enriched gene set annotations of genes within Simmental outlier RAiSD windows.

**Supplementary Table 23.** Significantly enriched QTL classifications from nSL loci in Red Angus.

**Supplementary Table 24.** Significantly enriched QTL classifications from RAiSD loci in Red Angus.

**Supplementary Table 25.** Significantly enriched QTL classifications from nSL loci in Simmental.

**Supplementary Table 26.** Significantly enriched QTL classifications from RAiSD loci in Simmental.


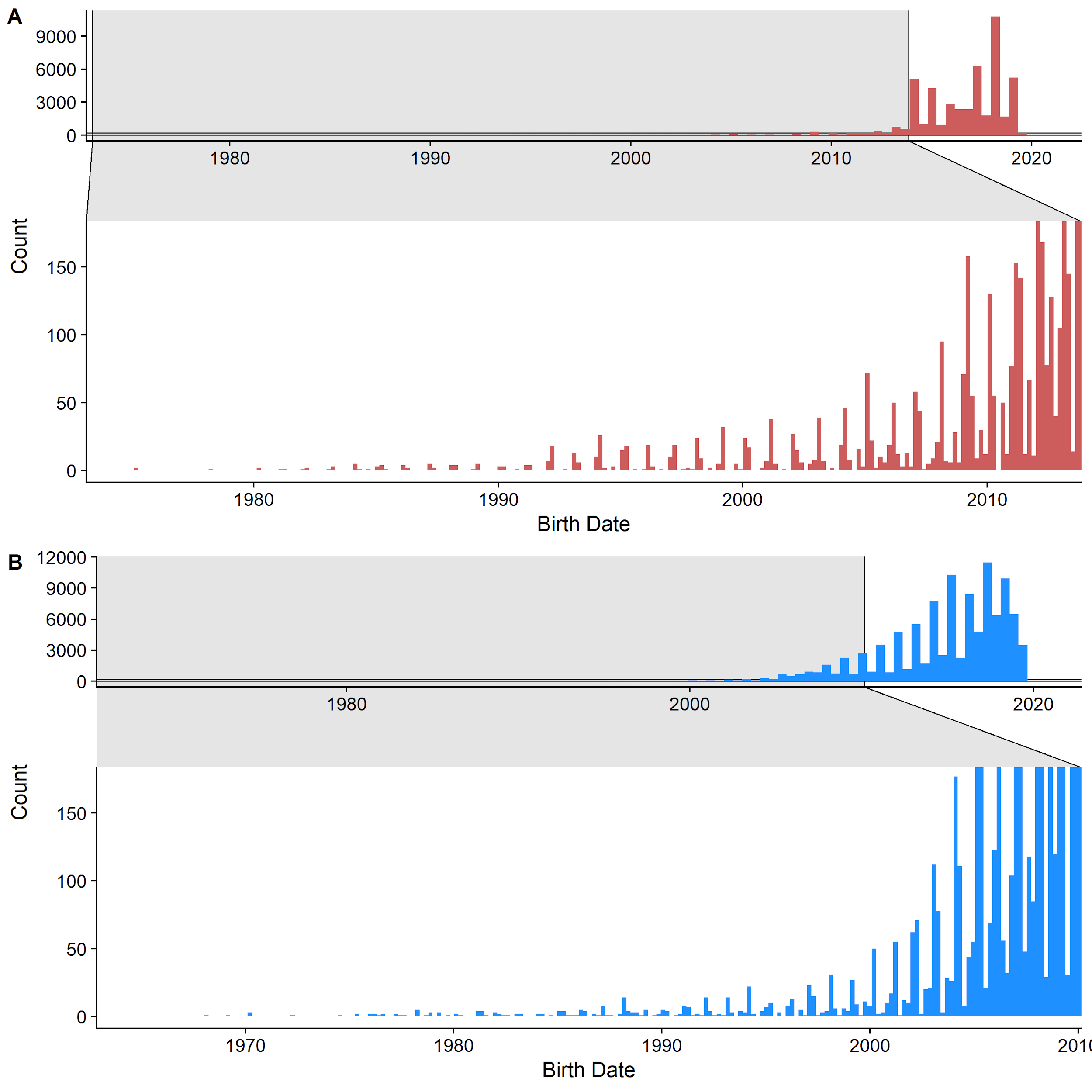


**Supplementary Figure 1. Birth date distributions of Red Angus and Simmental datasets.** Histograms of (A) Red Angus and (B) Simmental datasets. Left tails of the distributions (older animals) have been zoomed in the lower half of each panel.


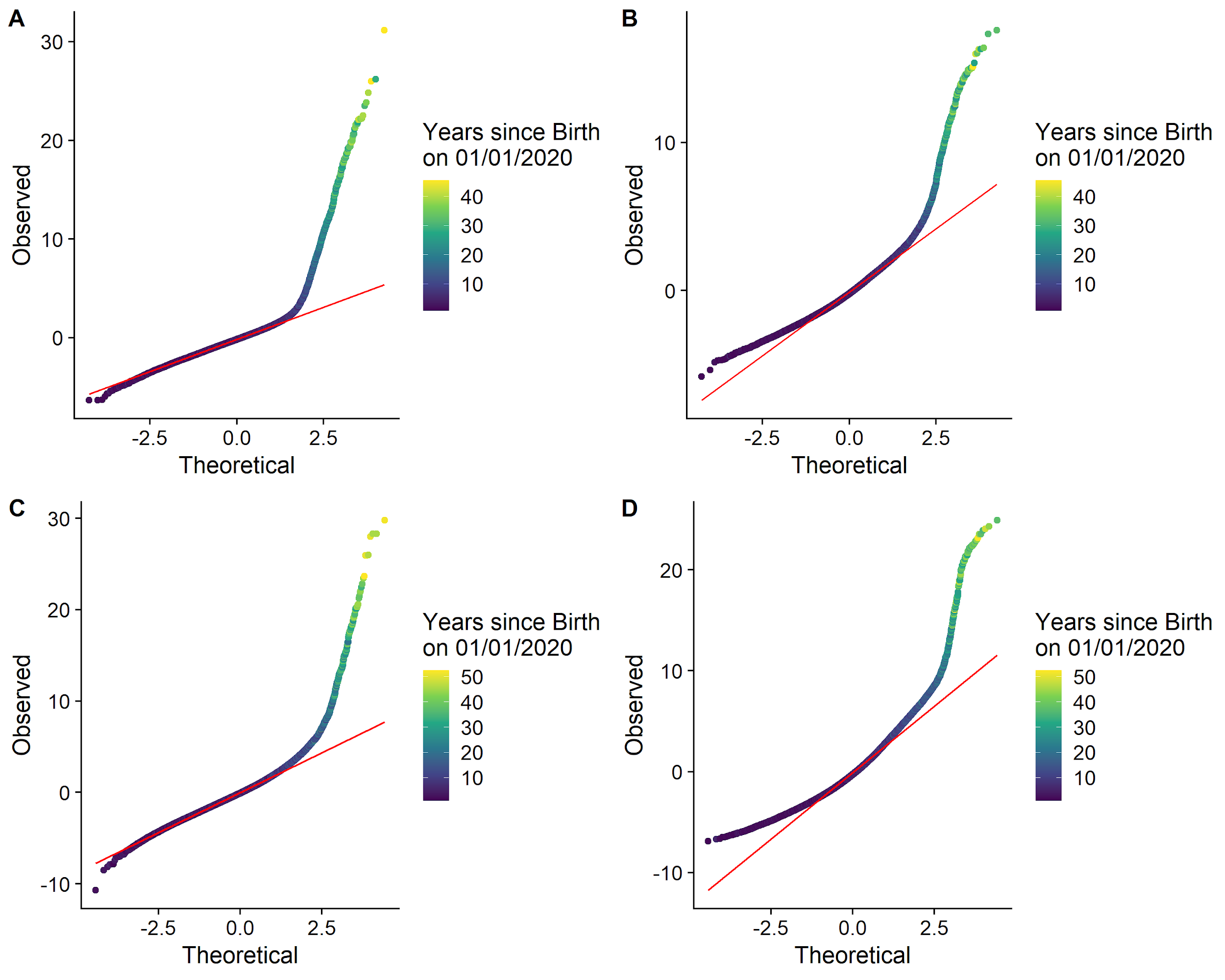


**Supplementary Figure 2. Q-Q plots from Birth Date GREML analysis.** Q-Q plots of GREML (A) residuals and (B) estimated breeding values for Red Angus individuals. Each point represents an animal, colored by the animal’s age as of January 1st, 2020. Simmental GREML Q-Q plots of (C) residuals and (D) estimated breeding values.


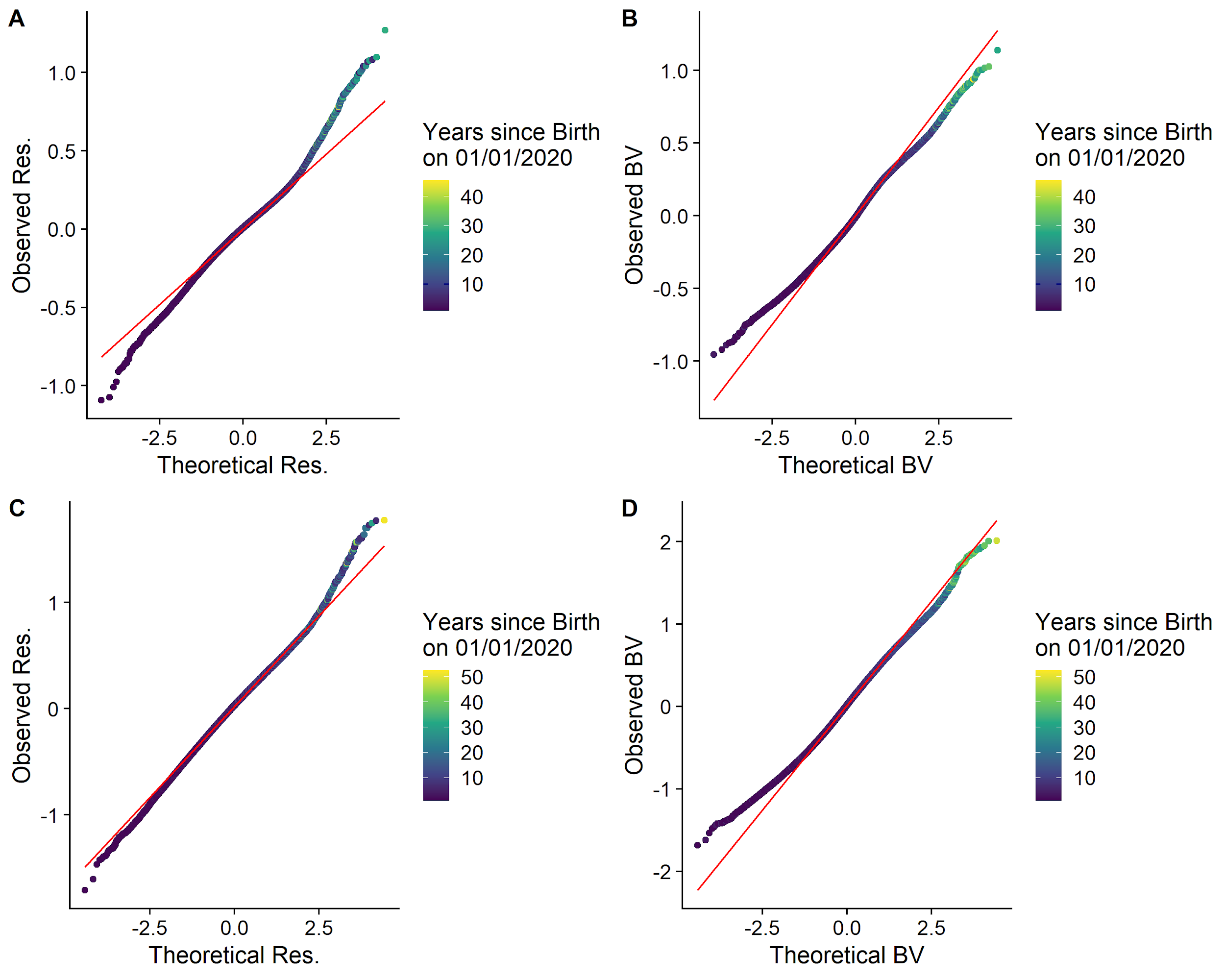


**Supplementary Figure 3. Q-Q plots from Box-Cox Transformed Birth Date GREML analysis.** Q-Q plots of GREML (A) residuals and (B) estimated breeding values for Red Angus individuals using Box-Cox transformed birth date as the dependent variable . Each point represents an animal, colored by the animal’s age as of January 1st, 2020. Simmental GREML Q-Q plots of (C) residuals and (D) estimated breeding values from analysis using Box-Cox transformed birth date as dependent variable..


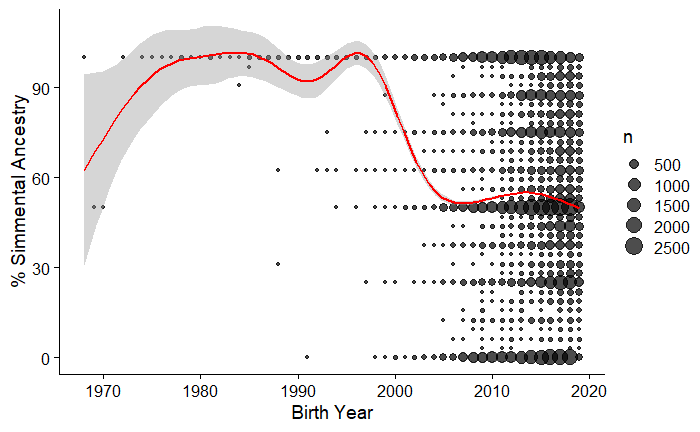


**Supplementary Figure 4.** The changing breed composition of registered Simmental. Counts of Simmental ancestry percentages over time in all genotyped animals in the American Simmental dataset. Points represent birth year/% Simmental ancestry combinations in the data, sized by the number of animals in each of those classes. The red line is a smoothed mean, surrounded by a 95% confidence interval in grey.


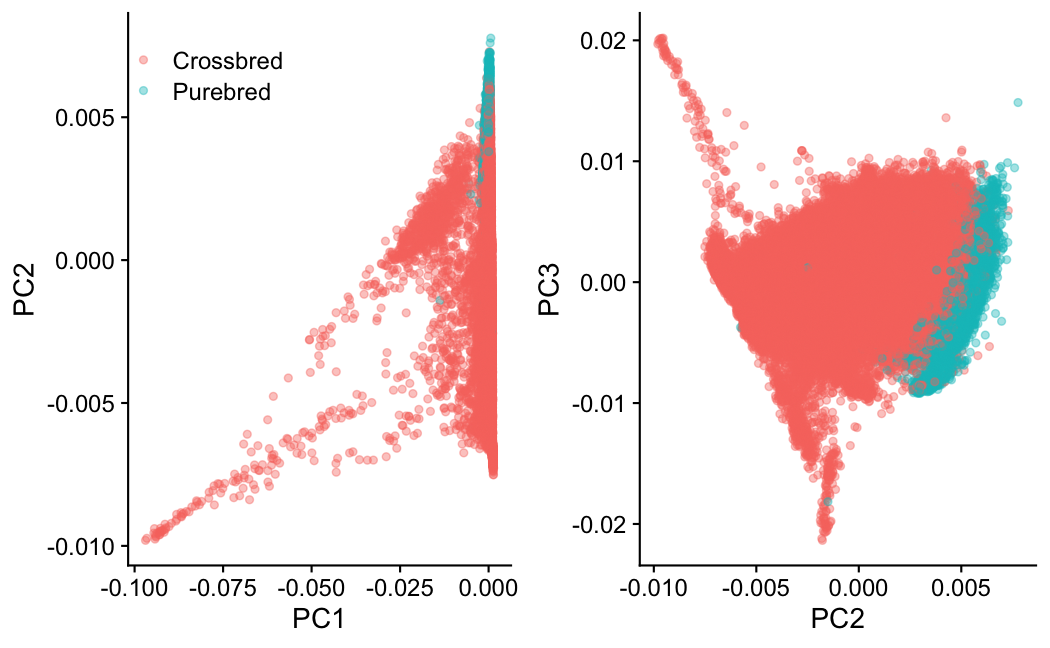
**Supplementary Figure 5.** Principal component analysis (PCA) plots for the Simmental Dataset. Purebred and crossbred animals in the herdbook are represented by blue and red points, respectively.
